# Parent–Infant Relational Health in a Disaster-Affected Region: A Qualitative Examination of Lived Experience and Perceived Impact of a Brief, Online Support Program

**DOI:** 10.3390/healthcare14121733

**Published:** 2026-06-16

**Authors:** Zoe C. G. Cloud, Nicole Paterson, Holly Foster, Tanudja Gibson, Shikkiah de Quadros-Wander, Anna T. Booth, Jennifer E. McIntosh

**Affiliations:** 1 The Bouverie Centre, La Trobe University, Melbourne, VIC 3056, Australia; z.cloud@latrobe.edu.au (Z.C.G.C.); n.paterson@latrobe.edu.au (N.P.); h.foster@latrobe.edu.au (H.F.); t.gibson@latrobe.edu.au (T.G.); s.dequadros-wander@latrobe.edu.au (S.d.Q.-W.); jenn.mcintosh@latrobe.edu.au (J.E.M.); 2 Werribee Mother Baby Service, Mercy Mental Health, Melbourne, VIC 3030, Australia

**Keywords:** infant mental health, parenting, psychoeducation, ecological systems theory, family stress

## Abstract

**Background/Objectives:** The family constitutes a primary ecological system shaping infant emotional and mental health. Parent responsiveness in particular shapes early regulatory capacities in the developing child. Added contextual stress such as that associated with natural disasters may strain caregiving relationships. Brief, universally accessible parenting interventions offer scalable support for strengthening early relational health and may be useful in contexts of natural disaster-related stress as well as in the general population. This qualitative study examined the perceived impact and contextual relevance of MERTIL (My Early Relational Trust-Informed Learning) *for Parents*, a brief digital psychoeducational parenting program targeting early relational health, among families raising young children in disaster-affected communities. **Methods:** Fourteen parents residing in the Hunter New England and Central Coast region of New South Wales, Australia, with young children aged 0–5 years, participated in semi-structured interviews conducted approximately 6 months after completing MERTIL *for Parents*. Interviews explored lived experiences of parenting in the context of natural disaster (analysed via applied phenomenological methods) and parents’ perceptions of program components that supported everyday caregiving (analysed via reflexive thematic analysis and content analysis). **Results:** Parents described interconnected personal, relational, and environmental stressors that influenced aspects of the parent–infant relationship. Key retained knowledge from the program included a normalisation of parenting challenges, a strengthened understanding of attachment, trust, safety and repair, and attuned, emotion-focused parenting practices. **Conclusions:** This pilot study illuminates the lived experience of parenting in disaster prone regions and highlights the potential for this brief, universal digital parenting program to provide support for early relational health in such contexts.

## 1. Parent–Infant Relational Health in a Disaster-Affected Region: A Qualitative Examination of Lived Experience and Perceived Impact of a Brief, Online Support Program

Alongside normative demands of caring for a young child, many parents navigate personal, relational, and contextual challenges that can accumulate and impact the caregiving relationship [1]. Population data indicate that a substantial proportion of parents experience mental health difficulties during the early parenting years [2], while many also bring their own histories of adversity, neurodevelopmental differences, and relational or intergenerational trauma [3]. These factors do not operate in isolation; rather, they layer across ecological systems to shape the conditions under which parent–child relationships develop [1,3]. From a bioecological perspective, child development is embedded within nested environmental systems ranging from immediate interaction with their home environment (e.g., with caregivers) to broader social and environmental contexts [4,5,6,7]. Within this framework, the parent–child relationship represents the most proximal developmental environment, while wider structural/societal influences (e.g., socioeconomic pressures, community resources and environmental instability) form the context, or outer layers, that influence relational health both indirectly [4] and bidirectionally [8].

Increasingly, families in Australia are parenting within geographic regions affected by recurrent natural disasters and climate-related events [9,10], adding further complexity to an already demanding developmental period. Exposure to natural disasters such as climate change-related events is linked to significant mental health impacts for both adults and children, with one in four Australians directly affected by climate-related events meeting screening criteria for post-traumatic stress disorder [11]. Children exposed to bushfires, floods, or other climate-related events may experience anxiety, trauma-related symptoms, and behavioural dysregulation [12,13]. As bioecological models emphasise that children’s responses to environmental stress are shaped primarily by proximal caregiving environments [14], parenting processes can buffer or amplify the impacts of stress, with sensitive, attuned caregiving serving as a key protective factor [15].

Importantly, disaster exposure rarely represents a single discrete event. Family-systems research shows that exposure to multiple concurrent stressors can disrupt family functioning when coping resources are strained [16], and can further compound inequities [17]. In disaster-affected communities, environmental disruption often intersects with economic uncertainty, disruption to education, health and other social services, parental mental health challenges, physical endangerment, loss of critical resources, and ongoing recovery demands [18]. More frequent disasters pose an ongoing challenge to families’ sense of safety and stability. Together, these experiences resemble “compounding fractures” within the early relational environment, where normative parenting challenges are layered with additional pressures that can test parental capacity for emotional regulation, reflective functioning and relational attunement.

Interventions for early relational health at the family level following periods of disaster exposure are therefore warranted [16]. Strengthening the early parent–child relationship may represent a scalable pathway for supporting child development and family wellbeing in these contexts, particularly when supports are relatively accessible, low-burden, and can reach families before difficulties become entrenched.

### 1.1. Supporting Relational Health

Early childhood development unfolds within relational systems, with the quality of daily parent–child interactions shaping trajectories of socioemotional health, stress regulation, and later adaptation [19,20,21]. Relational health in the infant-parent dyad includes parental security, trust, responsiveness, and sensitivity as foundational processes that scaffold attachment organisation and emotional development [19,22]. When caregiving environments are disrupted or overwhelmed, relational trauma may emerge through persistent breakdowns in co-regulation and emotional safety [23,24]. Crucially, core components of relational health are modifiable through intervention [25], rationalising that non-context-specific universal approaches can offer early support, without requiring difficulties to reach clinical thresholds.

Universal parenting interventions have traditionally been delivered via psychoeducational workshops or facilitated groups (e.g., Circle of Security); [26] and have demonstrated benefits for parental self-efficacy, wellbeing, and positive parenting practices, while reducing child internalising and externalising symptoms [27]. However, structural barriers can limit access to in-person and/or facilitated groups, particularly for families living in regional or disaster-affected communities, including limited service availability, workforce limitations, costs, and inflexible delivery times or formats [28,29]. Families also encounter practical obstacles, such as feeling time-poor, juggling competing caregiving demands, transport, and stigma, all of which can reduce enrollment and retention [28,29]. These inequities became especially magnified during the COVID-19 pandemic, when service disruptions coincided with heightened parental stress and child vulnerability [30].

Digital parenting programs offer a scalable alternative capable of reaching families within complex and changing contexts, such as in the wake of natural disasters. Online self-directed programs have been shown to improve parenting confidence, reduce parental distress, and support positive parent–child interactions [31,32,33]. Despite this growing evidence base, the suitability of brief online parenting programs for families experiencing stress in contexts of natural disaster has not been well tested.

### 1.2. My Early Relational Trust-Informed Learning (MERTIL) for Parents

MERTIL (My Early Relational Trust-Informed Learning) *for Parents* is a brief, universally accessible Australian online parenting program designed to strengthen early relational trust and prevent relational trauma for parents of children aged 0–5 years [34,35]. The program delivers concise, psychoeducational video content aimed at enhancing parental insight into infant emotional needs, trust-building behaviours, and the relational foundations of development. Its digital format is designed to reduce common access barriers and enables delivery at a broad community level, including in geographically dispersed regions.

A recent pilot evaluation conducted through metropolitan family and maternal child health services found high acceptability for MERTIL *for Parents*, with parents endorsing the program’s clarity, brevity, and emotional tone [34]. Preliminary three-month outcomes indicated improvements across multiple evaluation domains, including parenting confidence and competence, relational enjoyment and attunement, openness to help-seeking, and reduced irritability toward the child [34]. While these findings support the program’s feasibility and short-term effectiveness in a metropolitan context, it remains unclear whether similar benefits are perceived and sustained among families experiencing stress in the context of natural disaster.

In the Hunter New England and Central Coast (HNECC) region of New South Wales, Australia, families with young children have navigated successive natural disasters, including bushfires, floods, and severe weather events alongside the broader disruptions of the COVID-19 pandemic. For many families, these events are likely to have compounded existing socioeconomic, relational and psychosocial pressures, adding further strain to the already complex task of early parenting during a developmentally sensitive period. Within these contexts, caregiving relationships may be both vulnerable to stress and uniquely positioned as sources of stability and recovery. Given evidence that parenting quality can buffer the impacts of environmental adversity on child mental health and neurodevelopment, evaluating accessible, relationship-focused supports that can reach families amid the stress of natural disaster is both timely and necessary.

### 1.3. The Present Study

This pilot qualitative study extends prior evaluations to describe lived experiences of early parenting and family wellbeing in the disaster-affected areas of the HNECC region, and to examine the perceived impact and relevance of MERTIL *for Parents* in this specific setting. Using in-depth qualitative interviews with parents who had completed the program, the study aimed to:

1. Explore the lived experience of parenting young children in a disaster-affected region.
2. Articulate whether components of MERTIL *for Parents* enabled parent capacity and confidence within this context.
3. Examine the application of insights, if any, from MERTIL *for Parents* in everyday caregiving.

## 2. Method

### 2.1. Study Design

A three-phase approach to this exploratory qualitative study was structured to (1) provide an in-depth account of parenting in a disaster-affected region and describe (2) retained program value and (3) real-world application several months post-completion. Therefore, an inductive analysis, informed by modified empirical phenomenological methods and the theoretical framework of bioecological systems, was used to understand the lived experience of parenting in a disaster-affected region (Domain 1), followed by reflexive thematic analysis and qualitative content analysis to examine retained program value and applied learning across the full sample (Domains 2 and 3 respectively).

### 2.2. Researcher Characteristics and Reflexivity

Data collection and analysis were led by two researchers: ZC, a research fellow and psychologist and NP, a research assistant and social worker. Both researchers had experience in working with individuals from vulnerable cohorts and were able to conduct interviews in a trauma-informed manner. Researchers maintained reflexivity through the process by remaining conscious of their personal and professional knowledge and values and ensuring that these did not interfere unduly with the research process. Neither of the researchers who collected and analysed data were involved in the original development of MERTIL *for Parents*, but were familiar with its general structure and components and have previously published on a pilot evaluation of the program.

### 2.3. Participants and Context

Participants comprised a nested sample of 14 parents who had participated in a wider dissemination project for the MERTIL *for Parents* program. While the sample size was not determined a priori, it was deemed appropriate for the selected analyses and is consistent with those drawn on in prior studies employing similar qualitative methodologies [36]. The study had an original target sample of 25 but due to slower than anticipated recruitment, likely due to the competing pressures faced by the eligible population, we elected to halt recruitment in order to progress with the study timeline once a suitable minimum sample had been achieved. Participants had initially been referred to the MERTIL *for Parents* program by their Maternal and Child Health (MCH) nurse.

Subsequent to a survey-based study of 201 parents, eligible participants were invited to self-select into this follow up study. Participants were eligible if they had completed both the MERTIL *for Parents* program and pre- and post-program surveys, and they had elected via these surveys that they were interested in being contacted regarding future research opportunities. They also had to meet the following inclusion criteria: over 18 years old, had child/ren aged 0–5 years, resided in the HNECC region of New South Wales, Australia, and were comfortable completing program material and surveys in English. A total of 51 eligible participants were invited to self-select into the follow up study, with a final sample size of 14 participating parents, including 13 mothers and 1 father.

All parents in this sample reported sharing their parenting with their child’s other parent. Table 1 displays further demographic information provided by parents at baseline (i.e., prior to completing the MERTIL *for Parents* program). Parents were remunerated for their interview time, receiving a $100 e-gift card.

As seen in Table 1, almost all parents were aged under 45 years (92.9%), spoke English at home (100%) and were the parent of one child (79.0%). One parent identified as First Nations (7.1%). All but three parents (21.4%) reported experiencing a range of contextual family stressors, with an average of 2.8 different stressors per family (*SD* = 2.50, range: 0–7). Over half of all parents reported that their family stressors impacted on their parenting at least some of the time (57.1%).

All 14 participants contributed data to analyses for Domains 2 and 3. A subset of seven interviews containing the richest descriptions of parenting in the context of natural disaster were selected for in-depth inductive analysis in Domain 1 to understand the lived experience of parenting in this context. In other words, we elected (a priori) to draw on half of the sample to answer the research question for the Domain 1 depth study. Conferencing between the two main coders, with input from a third senior qualitative researcher, determined the 50% of cases that were selected.

### 2.4. Intervention

MERTIL *for Parents* is an online program accessed via the internet and can be viewed on any device (i.e., phone, computer, or tablet). The primary component of MERTIL *for Parents* comprises 40 min of narrated and animated video material, consisting of four ‘chapters’, each approximately ten minutes in duration (Table 2). A detailed description of the program has been published elsewhere [34,35].

### 2.5. Interview Protocol

Consenting participants were interviewed 6 months (on average) after completing the MERTIL *for Parents* program (*SD* = 3 months). This was designed to be a sufficient passage of time post-intervention for parents to practice, sustain, and reflect on the application of program knowledge. Interviews were conducted via Zoom, between September and October 2025, and were approximately 30–45 min in duration. Interviews began with rapport-building and standardised ethical disclosures, including assurances of confidentiality, voluntary participation, and clarification of limits to confidentiality in the event of safety concerns. Participants were invited to ask questions before proceeding. The interview explored three main themes: Parenting in a disaster-affected region (Domain 1); retained program value and real-life application (e.g., “*What, if anything, has stayed with you from completing the program?”, “Can you think of any examples where you applied something you learnt in the program to your everyday life as parent*?”); usefulness of MERTIL *for Parents* for disaster-affected parents (e.g., “*Were there any parts of the program that felt especially relevant given your experience of parenting through disaster recovery*?”). Interviews were audio-recorded and transcribed by Zoom, and then checked for verbatim and accuracy by the researchers.

### 2.6. Procedure

Practitioners from family and maternal health services in the HNECC region were invited to recommend the MERTIL *for Parents* program to parents during their telehealth or in-person appointments. Interested parents were given free access to the program and downloadable support materials via a QR code on promotional materials or bespoke link supplied by their practitioner. At program enrolment, parents were invited to opt in to participate in follow-up research. The research team periodically extracted enrolment and engagement data from the program’s online hosting platform. Following a rolling recruitment strategy, parents were contacted about an interview 36 months after completing the program. Data were screened for program and questionnaire completion, parent and child age, and postcode to confirm residency within the HNECC region.

Parents who lived in HNECC, completed the MERTIL *for Parents* program, and consented to research contact were invited via email to participate in a paid follow-up interview. Email invitations contained study information and a link to an online consent form (hosted by QuestionPro). Parents were contacted up to three times and could decline or withdraw at any stage. Following written consent and nomination of availability, interviews were scheduled directly with research staff and conducted via Zoom version 6 (approximate duration 30–45 min).

### 2.7. Ethical Considerations

The study was approved by La Trobe University’s Human Research Committee (HREC:25332). Participation was voluntary, all participants provided written informed consent and were advised that the interview could be terminated at any time. All identifying information was removed from the transcript prior to analysis.

### 2.8. Analytic Approach and Methodological Integrity

**Domain 1: Parenting under natural disaster-related stress.**

To understand the lived experience of early parenting in the context of stress related to natural disaster, a subset of seven interviews that contained the most experientially rich descriptions were selected as they lent themselves to analysis within an inductive frame, informed by empirical phenomenological methods [37,38]. Analyses sought to explicate and describe the core constituents of parenting in a disaster-affected region which included recent bushfires, flooding, and post-pandemic economic strain. Using a phenomenological coding approach, the relevant section of each transcript was sectioned into raw meaning units, representing self-contained constructs. Each meaning unit was then transformed to elicit its subjective meanings, and the themes for each participant were summarised into individual statements of lived experience. One independent coder (ZC) conducted multiple readings of the relevant passages for familiarisation, chunking of meaning units, transformation and synthesis. Cross researcher validation was ensured through cross-coding of 4 transcripts by a second independent coder (NP), with coding disputes or new insights discussed and resolved via conferencing. This phase established a contextual foundation for understanding program relevance and application. To understand how these experiential themes operated across multiple ecological levels, themes were subsequently interpreted through a bioecological lens [4,5,6,7,8].

**Domains 2 and 3: Retained Knowledge and Real-Life Application.**

The relevant data from all 14 participants were analysed using the complementary approaches of reflexive thematic analysis and qualitative content analysis, respectively [39,40,41,42]. The reflective thematic analytic approach was selected given its appropriateness for studies of complex and subjective experiences, and a briefer content analytic approach selected for its utility with the available information about real-life application of program knowledge. These approaches were selected based on their overall appropriateness for identifying patterned experiences across parent accounts while preserving attention to context and variation.

For Domain 2 coding, the data were subjected to the following six phases [41,42]: (i) familiarization with the data through reading and re-reading; (ii) coding (generating short labels for features across the dataset); (iii) generating initial themes; (iv) developing and reviewing themes, (v) refining, defining and naming themes, and (vi) writing up (constructing the final analysis and including quotes).

For Domain 3 coding, we subjected data to a qualitative content analysis to understand patterns in the data. Using a standardised coding table, one coder extracted and coded transcript data. Codes and themes were reviewed by a second independent coder. Data analysis occurred across four stages: (a) decontextualisation (deconstruction of the transcript into meaning units); (b) recontextualisation (identification of codes); (c) categorisation (identification of themes); and (d) compilation (drawing of group-level conclusions and variations, with conferencing between coders and checking of the original text).

Transcripts were analysed until a point of thematic sufficiency was met; that is, no new coding themes or subcategories were revealed in parental experiences within our sample. It is recognised that further interviews with a larger sample may have revealed additional unique experiences or nuances. Analysis of the study’s sample specifically proceeded through iterative familiarisation, coding, theme development, refinement, and cross-coder verification (ZC and NP), with coding disputes resolved via conferencing with JM.

## 3. Results

**Domain 1. Parenting in a disaster-affected region (*N* = 7)**

**Figure 1:**
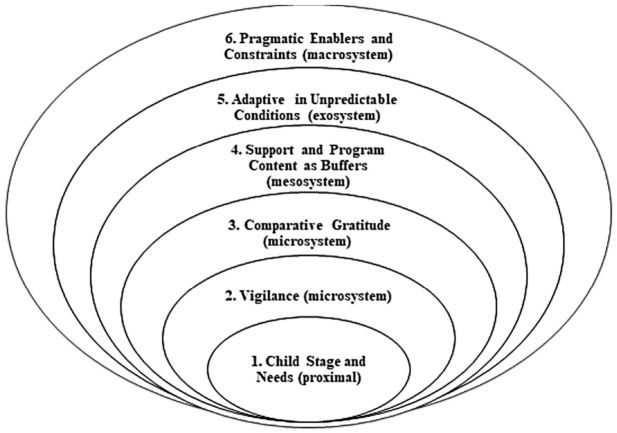
Ecological Mapping of Parenting Experiences in a Disaster-affected Region.

Here, we present an overview of the shared themes derived through a modified phenomenological method, describing core and shared components of how parents understood, navigated, and made sense of parenting under conditions of disaster-related stress. Across the seven interviews, six overarching experiential themes were identified. Figure 1 presents an ecological mapping of parents’ experiences, illustrating how parenting challenges were distributed across nested systems ranging from proximal caregiving interactions to broader environmental and structural contexts.

**Theme 1. Parenting Experience Shaped by Child Developmental Stage and Needs.**

The extent to which natural disasters disrupted parenting depended partly on the child’s age and needs. Two parents in the subsample described feeling protected from major disruption due to their children being young infants and primarily at home with less reliance on external activities, e.g., “bub was very young at that time, so he doesn’t need to be out there as much” (P26). Another parent of a young infant additionally noted the potential financial impact of weather events occurring during this developmental stage, e.g., “the financial impact of having a baby and you’re off work and then having to replace things out of the blue” (P13). In contrast, three parents of more active infants or toddlers, or older children, described greater limitations when weather restricted access to playgroups, outings, or social activities, e.g., “there are not a lot of things in [location] that you can do that are indoors-related. We rely very much on parks and things here” (P10).

One parent described having a medically vulnerable infant which compounded complexity, as adverse weather intersected with reduced access to health services or specialised care (P24). As illustrated in Figure 1, these experiences primarily reflected proximal microsystem processes [5,6], where child developmental needs shaped how broader environmental disruptions were experienced within daily caregiving. In another example, one parent shared they “had no electricity for 48 h, and it was just me and my one-year-old… had to problem solve, think outside the box to get through” (P2).

**Theme 2. Emotional and Temporal Vigilance.**

Five of the 7 parents in this subsample described heightened attention to weather patterns, community alerts, social media updates, and the possibility of future events. All seven parents referred to a vigilance that reflected the blending of past experiences (such as previous floods, e.g., “after 48 h [without power or services], it starts to get a little bit really scary” (P2)), present environmental cues (ongoing rainfall, saturated ground), e.g., “I find wet weather makes me anxious” (P10) and anticipated future risks (concerns about climate change, repeat events, or ongoing infrastructure vulnerability, e.g., “with climate change, you know, the long-term projections are pretty dire” (P1). While sometimes stressful or anxiety-provoking, parents emphasised their efforts to remain emotionally present and regulated for their children. One parent explicitly noted that an increased focus on weather necessitated a decreased focus on parenting, sharing: “every time we know that there is rain coming, it takes away from parenting when your focus has to be on making sure the household is safe” (P10). This dual focus on monitoring threat while maintaining caregiving presence was a central feature of the lived experience of parenting in a disaster-affected region.

**Theme 3. Framing Parenting Struggles Against Comparative Gratitude.**

Across all interviews, parents situated their experiences within a comparative frame comparing themselves to others who were more severely affected by floods, storms, or disruptions. Their comparison to others generated feelings of luck, gratitude, and sometimes humility. This shaped how parents assessed their own stress, with many minimising their challenges because “others were more severely impacted”, with descriptions of their experiences of natural disasters including: “traumatic, but no lasting impacts like others” (P24),”not as bad as others” (P26), or “a lot of people had it way worse than us” (P10).

**Theme 4. Relational Support and Program Content as Buffers.**

Six of the seven parents emphasised the protective value of social support, parenting groups, and insights from the MERTIL *for Parents* program. Importantly, parents reported that the MERTIL *for Parents* program helped them to stay attuned to their child in crisis or under pressure, promoting normality and safety and buffering their child from stress, e.g., “when I was parenting through floods, just trying to keep everything as normal as possible for her. So she’s not being impacted by the actual fear and stress of it” (P2). Further, parents described the applicability of trauma concepts from the program to families exposed to natural disasters, in healing and recovery post-crisis, and the promotion of help-seeking behaviour when more support was needed. Alongside face-to-face community supports, the MERTIL *for Parents* program appeared to assist parents regulate their emotions, normalise their experiences, and maintain connection with their children even when external conditions were challenging. Given weather sometimes restricted access to in-person parenting or support groups, parents described MERTIL *for Parents* as meaningful and helpful in navigating stressful periods.

**Theme 5. Adaptive and Flexible Parenting in Unpredictable Conditions.**

All parents described engaging in ongoing adaptation to changing weather conditions, service disruptions, and unpredictable routines. Flexibility became both a practical and coping strategy when parents needed to modify plans, stay indoors, reorganise childcare or appointments, or adjust expectations about what their parenting experience would be like. For some, this felt manageable and simply paralleled the normative flexibility required in early child rearing. For others, particularly those isolated or indoors for long periods, it led to loss of access to valued activities (e.g., social connection with other parents/families), with one parent sharing the additional financial impact of this: “going a bit stir-crazy indoors all the time, not being able to go out and do stuff…most of the indoor stuff you have to pay for” (P13). Parenting under these conditions required daily problem-solving, tolerance of uncertainty, and considerable emotional energy.

Themes 2–5 collectively demonstrate interactions between microsystem and mesosystem processes (Figure 1; [5]), where parental emotional regulation, social supports, and program engagement mediated the translation of environmental stress into everyday parenting experiences.

**Theme 6. Pragmatic Enablers and Constraints (Geography, Services, Socioeconomic status).**

Parents’ sense of safety and stability was enhanced by environment, infrastructure, and socioeconomic contexts. Three parents explicitly described living on “higher ground”, in newer housing, or in well-serviced areas as a buffer that reduced perceived risk. Conversely, parents who experienced service disruptions (e.g., power outages, road closures, or limitations to their access to services) or who described living in housing vulnerable to natural disasters shared experiences of greater strain, e.g., “when the main road into your little town gets cut off because it’s basically a bridge… we are cut off from main services.” (P2) Socioeconomic resources further shaped what parents could access, whether they could absorb unexpected costs, and how constrained they felt when weather restricted movement or activities. For all, these contextual conditions formed the backdrop against which parenting decisions were made and infant relational challenges emerged. These contextual influences correspond to exosystem and macrosystem levels within the ecological model (Figure 1; [5], highlighting how structural and geographic conditions shaped perceived safety and parenting capacity. As one parent described, “we have issues in the downstairs of our house. So every time we do have a downfall, it is very stressful in our household … I would say, personally, it probably does impact my parenting” (P10).

**Domain 2. Retained Program Knowledge**

Through reflexive thematic analysis, five interrelated themes describing parents’ retained knowledge from the MERTIL *for Parents* program were identified. As seen in Table 3, parents generally found the program to be supportive, practical, and personally meaningful, and felt it offered both foundational concepts and actionable strategies that continued to inform their caregiving approach beyond program completion. Table 3 presents relevant quotes from interview transcripts to illustrate the detail of each theme.

**Domain 3. Real-Life Application of Program Learnings**

Findings from the content analysis describe the contexts in which parents applied new skills, the relational processes they enacted, and the perceived benefits for both child and parent.

**Contexts of Application**

Parents most commonly described applying program concepts during routine but emotionally charged caregiving moments. Two primary contexts were identified. First, everyday separation–reunion cycles provided frequent opportunities for application of skills. Parents described brief separations associated with daily tasks (e.g., “you’ve got to get something done… you can’t kind of leave them unmonitored… I might put him in the cot … And of course, he gets upset” (P1)), during which infant distress prompted intentional use of program-informed responses (P1, P13, P19, P26). Second, parents applied learning during moments of emotional strain or “micro-ruptures,” when parental stress or concurrent demands competed with the child seeking their attention (P2, P13, P10, P20, P24), e.g., “If our mental health is impacted, and our parenting kind of not declines, but is impacted by it, that’s going to impact the children” (P10) In these situations, parents described pausing and engaging in deliberate repair rather than reacting impulsively. One parent reported extending program principles beyond the parent–child dyad by sharing knowledge with other caregivers to support consistency in caregiving approaches (P7).

**Application of Program Knowledge**

Parents described a central shift whereby they came to interpret child behaviour as communication of need rather than intentional misbehaviour (P2, P7, P8, P13, P14, P20, P22; e.g., “my husband and I are all like ‘she’s probably crying because she’s got an unmet need’, or, you know, ‘what would that need be, and how do we lean into that?’”(P7) This reframing was perceived to lead to more responsive caregiving, particularly during distress. Parents also described intentional practices of attunement and repair, including returning to the child following separation or conflict, providing comfort, and re-establishing emotional connection through calm and responsive interaction, including co-regulation (P1, P10, P13, P14, P16, P20, P24, P26). Importantly, parents reported increased tolerance of infant distress, recognising brief upset as developmentally normative rather than requiring immediate elimination (P1, P20, P26). Participants also described monitoring their own emotional responses, separating personal frustration from the child’s needs, regulating themselves prior to responding, and removing the pressure to respond perfectly, instead prioritising presence in the moment of repair (P10, P13, P20, P24; e.g., “[baby] cries a lot because he doesn’t want to sleep… he just needs some help to get to sleep. That’s separate from how I’m feeling.” (P13).

**Perceived Benefits for Children and Parents**

Parents perceived these changes as relevant to both child and parent functioning. Infants were described as calming more readily, reconnecting more quickly following distress, and appearing increasingly secure during separations (P1, P2, P19, P26). Parents also reported changes in their own caregiving approach, including greater intentionality, calmer responding, and increased confidence in managing emotionally challenging interactions (P2, P16, P20, P24, P26). Program participation was experienced as validating existing parenting instincts while providing a clearer framework for responding to everyday challenges (P7, P9, P10, P26), including reminders to openly express love toward their child (P8). Several parents noted that accessing the program early in their parenting journey supported integration of these practices into emerging routines, with flexible online delivery enabling engagement alongside caregiving demands (e.g., during contact napping).

Overall, findings from this domain indicate that parents’ learning was operationalised through small, repeated relational interactions embedded within daily caregiving activities. Parents described intentionally applying relational and attachment-based program principles to navigate separations, repair relational disruptions, and regulate their own responses. Parents reported that these strategies supported their connection with their child and strengthened their perceptions of their child’s emotional regulation. Where parents articulated these principles were not necessarily new knowledge, they reiterated that program content reinforced and validated their existing knowledge and practice of relationally oriented parenting.

## 4. Discussion

The present study explored parents’ experiences of engaging with a brief, universally accessible psychoeducational parenting program while living in and managing the stress of a natural-disaster-affected region. Participants described parenting as occurring within layered personal, relational, and contextual pressures. They felt that MERTIL *for Parents* was useful for managing the stress associated with their broader parenting context, as it offers an accessible relational framework that supports their reflection and connection in everyday caregiving interactions, bringing the focus back to the immediacy of parenting. The program was commonly described as providing a conceptual scaffold through which parents interpreted their child’s behaviour and their own responses. Overall, the findings indicate that participants found MERTIL *for Parents* to be meaningful and usable under conditions of complexity and contextual stress. Importantly, parents’ accounts suggested that their new learning was beneficial through application in ordinary caregiving moments, while environmental crises and associated stressors sat in the background to the ‘everyday-ness’ of parenting small children. This suggests MERTIL *for Parents* may have utility across various settings, and more broadly suggests that relationally oriented supports may operate through their impact within everyday family processes that underpin child and parent wellbeing.

### 4.1. Parenting Amid Contextual Challenge: Extending a Bioecological Understanding

Parents’ descriptions of their experiences align with bioecological perspectives of development, in which child wellbeing emerges through ongoing interactions between proximal caregiving relationships and their interconnection with broader ecological systems [4,5,6]. Participants rarely described the recent environmental disasters or associated financial stressors as isolated determinants of parenting difficulty; instead, these experiences appeared to frame or intensify existing developmental and relational demands associated with early parenting. Aligned with a family-systems perspective [16], findings suggest that stress accumulation, rather than single adverse events, shaped family functioning through the stretching of already over-extended resources available to cope.

Within this context, relational processes appeared to function as an important interface between wider social conditions and children’s emotional experiences. Parents interpreted the program content primarily through the lens of everyday caregiving challenges (e.g., responding to distress, managing separations, or navigating moments of parental frustration). Echoing broader discussions within family and child mental health research, proximal relational processes may buffer or mediate the effects of ecological stressors on developmental outcomes [8,43,44]. Importantly, environmental adversity did not have a singular impact on parenting experiences for those in the current study. Rather, adversity was felt to compound normative parenting challenges, reinforcing the importance of multi-tiered supports relevant to families experiencing intersecting levels of risk. As with recent review evidence [45], our findings centre relational health within families and embedded social systems as having potential to be active drivers of protective impact.

### 4.2. Value of MERTIL for Parents Within Contexts of Challenge

Parents’ accounts suggest several ways in which MERTIL *for Parents* may have been experienced as supporting their caregiving in a disaster-affected region. Parents frequently described a relational reframing of child behaviour, viewing distress signals (e.g., crying, whining) as communication rather than misbehaviour, alongside increased self-compassion and awareness of their own emotional responses. These shifts were often linked to small, practical adjustments in everyday interactions rather than structured behavioural change. Indeed, the program aims to support parental reflective functioning and attunement, processes considered central to early relational health [46,47]. Notably, parents did not typically describe the program as adding additional parenting demands; instead, it was experienced as simplifying or validating their understanding of relational wellbeing. This distinction appears particularly relevant in the current context where families experienced competing pressures across family, community, and environmental systems.

Given the qualitative and exploratory nature of the study, and its small sample size of self-selected participants, these interpretations should be considered provisional. Future research could establish further perceived usefulness of the program, testing the generalisability of these findings beyond the study’s relatively homogenous sample. Perceptions from parents in this study do, however, indicate the potential for brief psychoeducational interventions grounded in relational principles to complement existing family supports by strengthening proximal developmental processes rather than targeting isolated behavioural outcomes. Such approaches may align with broader family-focused prevention efforts aimed at supporting children’s emotional and psychological wellbeing through strengthening everyday caregiving relationships. Further, parents’ experiences in this study suggest that low-burden access and flexible digital delivery were perceived as compatible with the realities of early parenting under changing conditions.

MERTIL *for Parents* was designed to be universally applicable, and pilot findings reported here suggest that it is well suited to contexts of added environmental stress. It appears to provide a focus on the modifiable aspects of parenting in daily life, even for parents facing the additional stress and challenges associated with natural disasters, and may therefore be appropriate for families facing other contextually stressful events. It is important to note that many of the themes identified here (for example, co-regulation, emotional overload, relational repair, attunement and flexibility in parenting) could also emerge in various other contexts as well as for parents facing the challenges of natural disaster. The findings reported here may reflect an interaction between disaster-related stress and broader early parenting challenges rather than disaster-specific processes.

For parents in the current sample, the provision of support did not require problem identification or clinical thresholds. Instead, the program was offered to parents as an adjunct to be integrated into an already busy family life. This contrasts with more intensive clinical or disaster-specific interventions, which, while essential for some families, may be less accessible or scalable at a population level [16,18]. From a family systems and public mental health perspective, accessible psychoeducational programs could be considered a supportive and viable part of broader prevention pathways by supporting relational processes linked to overall child and family wellbeing across diverse ecological contexts [27,33].

### 4.3. Implications for Family and Child Mental Health Systems

Brief relational programs embedded within maternal and child health services, community parenting supports, and digital public health platforms may enable broad reach without requiring specialised clinical infrastructure. While this was not identified as a barrier to program access for parents in this study, it is acknowledged that families experiencing digital poverty or exclusion would require additional support to engage with such interventions. This could impact families living in certain regions (i.e., those in rural areas with inadequate access to high-speed internet) or in certain circumstances (i.e., unable to afford adequate technology devices or program access). Further consideration could also be given to the adequacy of a brief, online program (whether as a standalone intervention or in tandem with other services) for families during periods of acute trauma and crisis.

Importantly, findings support implementation in disaster-prone regions without requiring programs to be framed exclusively as disaster interventions. Instead, relationally focused universal supports may provide continuity across both normative and extraordinary parenting contexts, aligning with public health approaches that emphasise resilience-building within everyday systems of care.

### 4.4. Strengths, Limitations and Future Research

This study offers in-depth insight into parent experiences several months following program completion. The real-world implementation context and ecological diversity of participant experiences enhance the relevance of findings for community-based delivery. However, although appropriate for the selected methods of qualitative enquiry [36], the sample size was modest and comprised self-selected participants, potentially biasing findings toward parents who experienced the program positively. Interviews conducted with self-selected participants could introduce both recall and social desirability bias. We note that no participants shared negative experiences of the MERTIL *for Parents* program content; it is possible that this potential bias influenced participants’ self-reported experiences of the program or related parenting contexts. Outcomes reflected perceived changes rather than independently measured behavioural or developmental outcomes. Additionally, the study was conducted within a single regional Australian context, and the sample was fairly homogenous (i.e., with limited diversity of gender, cultural background) which may limit generalisability to other populations or service systems Finally, while steps were taken to minimise researcher bias in analysis as noted in the methods section, the potential for allegiance bias is noted.

Future research should examine longitudinal outcomes associated with brief relational psychoeducation in contexts of ecological stress, including whether gains in parental understanding and regulation translate into measurable child developmental outcomes. In particular, exploration of continuous-access or booster models may clarify whether periodic re-engagement enhances sustainability of relational change, including circumstances under which MERTIL *for Parents* might be perceived as beneficial in tandem with more specialised parenting interventions. Quantitative and mixed-methods studies across a larger and more diverse cultural and socioeconomic context would further strengthen understanding of universal parenting programs for early relational health.

## 5. Conclusions

Parenting young children occurs within layered ecological systems in which normative developmental demands intersect with broader social and environmental pressures. In the present pilot study, strengthening early relational health through brief, accessible psychoeducation appeared to be both feasible and meaningful for families in a natural disaster-affected region. Universal support for early relational health, such as that provided by MERTIL *for Parents*, may be considered a viable option for helping families sustain attuned caregiving across contexts of both everyday stress and broader adversity. This highlights the potential public health significance of early relational prevention approaches for child and family wellbeing.

## Figures and Tables

**Table 1:** Baseline Participant Demographic Information (*N* = 14).

Variable	*N* (%)
Age	
26–35 years old	5 (35.7)
36–45 years old	8 (50.0)
46-years old or above	1 (7.1)
First Nations	1 (7.1)
English spoken at home	14 (100)
Number of children	
1 child	11 (71.4)
2 children	3 (21.4)
Contextual stressors	
Parenting stress	7 (50.0)
Mental health	6 (42.9)
Feeling overwhelmed	5 (35.7)
Work stress	5 (35.7)
Relationship stress	5 (35.7)
Illness/disability	3 (21.4)
Financial problems	1 (7.1)
Lack of support	1 (7.1)
Housing problems	1 (7.1)
Feeling lonely/isolated	1 (7.1)
A traumatic event/s	1 (7.1)
Not having trouble with any of these things at the moment	3 (21.4)
These days, do these stressors affect your ability to be the parent/caregiver you want to be?	
Not at all/rarely	6 (35.7)
Occasionally	5 (35.7)
Sometimes	2 (14.3)
Often	1 (7.1)

**Table 2:** MERTIL *for Parents* Program Components.

Chapter	Title	Content
1	MERTIL for My Family	Introduces program A young child explains the construct of trust
2	Why trust matters	Explores attachment and trust in parenting, particularly in context of relational trauma Outlines realistic expectations (the “good enough” parent) Provides strategies for building trust and shared delight between parent–child
3	Trust and trauma; rupture and repair	Explores building relational trust through parenting predictability and nurturing secure attachments (the “trust dance”) Outlines concept of rupture and repair Explores impact of parental trauma and family violence
4	Becoming your best parent, with support	Program summary Importance of seeking and utilizing support Recognising the need for professional help

**Table 3:** Reflexive Thematic Analysis of Retained Program Value.

Theme and Description	Illustrative Quotes
**Reassurance and Normalisation of Parenting Challenges** Parents retained program messages that normalised common parenting challenges, reduced self-criticism and pressure to be perfect and validated “good enough” caregiving. The program reassured parents that relational security develops over time and supported renewed confidence, particularly for those navigating difficult early parenting experiences or recovery from adversity (e.g., premature baby, maternal mental health challenges, disaster recovery).	**P1:** “as a parent, you can be pretty hard on yourself sometimes, and I think a lot of parents feel a bit inadequate… But, you know, those words you used, like, good enough, like, you don’t have to be perfect, it doesn’t exist.” **P8:** “sort of puts a bit less pressure on you to be doing it right all the time, … because they’re not fully developed, and you don’t respond right all the time as well” **P20:** “when I had [daughter], I ended up getting postpartum psychosis and the program grounded me when I come back and I just felt a lot more confident in myself and to look after [daughter]”.
**Strengthened Understanding of Attachment, Trust, Safety and Repair** Parents retained an expanded understanding of how trust and emotional safety are built through everyday caregiving interactions. Parents described greater awareness of developmental processes, the lifelong significance of early relationships, and the role of repair following relational ruptures. The program also prompted reflection on parent’s own histories, fostering hope and agency in shaping different relational futures for their children.	**P1:** “[Program] helped to clarify and reinforce just how that, that secure bonding, how important it is, and… how it gets built over time” **P13:** “Being aware of your own triggers… separating that from your kid’s behaviour…If you’re being triggered from something in your past… they’re not doing it on purpose.” **P24:** “Sometimes the trust is built in the repair… it really resonated.”
**Attuned, Emotion-focused Parenting Practices** Parents described retaining practical relational strategies centred on emotional attunement, viewing behaviour as communication, and supporting co-regulation. Parents increasingly recognised the influence of their own emotions on their child, particularly during periods of stress or disruption. Further, the program seemed to have flexible application across diverse parenting contexts, including neurodivergent development and disaster-related stress.	**P2:** “it was amazing, actually, like, just looking at it—all behaviours as communication, that’s so true. I think I use a lot of the tools, even now, like co-regulating with my 5-year-old.” **P7:** “babies cry, and that’s normal, and whilst it is normal, it’s still communicating, and so how do you meet a baby’s need through that behaviour.” **P8:** “my daughter is on the spectrum… the course kind of helped me think a little bit more about being a bit more stable and secure in yourself, and how that flows through to the child” **P14:** “The program did really reinforce that it’s not a cry, it’s her asking for something, trying to communicate with me as best she can, so, to not get frustrated by that, or to not ignore that”
**Accessible, Practical and Engaging Learning Format** Parents consistently valued the short, visually engaging, strengths-based delivery format (including relatable language, embedded videos, and practical examples). The program was a trusted source of science-based parenting within an oversaturated information environment. The program felt achievable during early parenthood and accessible to a broad range of caregivers.	**P1:** “Videos made complex social interactions clear and transparent.” **P7:** “The language… relatable… for a wide range of people.” **P10:** “… the way it was spun was very positive, and it was very, diverse, so I felt like a lot of people could relate to at least some part of it. I thought it was really easily accessible. I really liked the graphics, I found the voice very calming.” **P13:** “Short… easily digestible… not a huge commitment.” **P24:** “Right amount of information… right length… right pace.”
**Reinforcement and Validation of Existing Knowledge** The program reinforced and organised knowledge parents already held intuitively or had previously encountered by validating existing caregiving practices, providing an explanatory framework, and increasing confidence in applying relational principles.	**P1:** the tools that the program equipped or emphasised… they’re important in a normal circumstance. I imagine they’re even more important when you’re dealing with something like a natural disaster.” **P2:** “Back to basics of attachment with your child.” **P21:** “just kind of reassures you that you’re on the right track, and gives a bit of a framework to how you already think about it” **P26:** “it’s good to kind of be reinforced and, like, yeah, more research-based, evidence-based information, and with examples”

## Data Availability

The data that support the findings of this study are available on request from the corresponding author, A.T.B. The data are not publicly available due to their containing information that could compromise the privacy of research participants.
